# lncRNA RMRP predicts poor prognosis and mediates tumor progression of esophageal squamous cell carcinoma by regulating miR-613/ neuropilin 2 (NRP2) axis

**DOI:** 10.1080/21655979.2021.1974656

**Published:** 2021-09-13

**Authors:** Zhen Xie, Shuai Liu, Shicheng Chu, Yiqun Liu, Bingtao Huang, Qingguang Zhang

**Affiliations:** Department of Thoracic Surgery, Binzhou Medical University Hospital, Binzhou, China

**Keywords:** lncRNA RMRP, esophageal squamous cell carcinoma, prognosis, progression, proliferation, migration, invasion

## Abstract

The RNA component of mitochondrial RNA processing endoribonuclease (RMRP) has been reported to play a role in the development of various human diseases. The clinical significance and biological function of RMRP in the progression of esophageal squamous cell carcinoma (ESCC) and the potential mechanism were investigated in this study.A total of 118 ESCC patients were included in this study. The expression of RMRP in ESCC was analyzed with the help of the polymerase chain reaction. The cell counting kit 8 assay was employed to evaluate the role of RMRP in cell proliferation, and its functions in cell migration and invasion were assessed by the Transwell assays. Meanwhile, the clinical significance of RMRP in ESCC was estimated with Kaplan-Meier and Cox regression analysis.RMRP was significantly upregulated in ESCC, which was associated with the lymph node metastasis status, the TNM stage of patients, and a poor outcome of ESCC patients. Moreover, RMRP promoted the proliferation, migration, and invasion of ESCC cells via regulating miR-613/NRP2.RMRP was involved in the progression of ESCC through regulating the miR-613/NRP2 axis, which provides a potential target for the treatment of ESCC.

## Introduction

Esophageal cancer is a common digestive tract malignant tumor with a low survival rate and high recurrence [1]. Among various subtypes of esophageal cancer, esophageal squamous cell carcinoma (ESCC) is one of the most common histological with high mortality [2]. Patients with ESCC are always diagnosed at an advanced stage due to the lack of typical symptoms in the early stage [3]. On the other hand, metastasis is one of the main factors that result in recurrence after surgical treatment, which leads to therapeutic failure. If the disease development can be predicted by the detection of relevant indicators in patients, the clinical prognosis of patients would be greatly improved. Thus, it is advantageous to appraise novel biomarkers to obtain more information on the progression of ESCC.

Long non-coding RNAs (lncRNAs) are a series of RNAs with a length of over 200 nucleotides and without the function of coding proteins [4]. lncRNAs have been demonstrated to mediate the expression of genes at the post-transcriptional or transcriptional level. Accumulating evidence indicated that lncRNAs, especially the dysregulated lncRNAs, might be involved in the pathogenesis and progression of human diseases [5]. LncRNA RMPM is a lncRNA located in the nucleus, nucleolus, and mitochondria, which has been widely disclosed to express in miscellaneous human diseases and play critical roles, such as bladder cancer, hepatocellular carcinoma, and gastric cancer [6–8]. RMRP could regulate miR-613 and therefore participate in the progression of non-small lung cancer and hepatocellular carcinoma [9,10]. miR-613 was identified as an indicator in the diagnosis and prognosis of ESCC [11,12]. In our previous studies, Semaphorin 3 F was revealed to act as a tumor suppressor of ESCC and involved in the lymph node metastasis development by regulating neuropilin 2 (NRP2), which is a direct target of miR-613 [13].

RMRP was speculated to be involved in the progression of ESCC through regulating NRP2. Therefore, the expression and function of RMRP and its interaction with NRP2 were evaluated in this study.

## Materials and methods

### Patients and tissues

This study had obtained ethical approval from the Ethics Committee of Binzhou Medical University Hospital (approval no. 2011–034) and all participators had signed informed consent. A total of 118 ESCC patients that underwent surgical treatment at Binzhou Medical University Hospital were included in this study from 2012 to 2015. The clinical features of the patients were summarized in Table 1. The ESCC tissues and adjacent nontumorous tissues were collected as previously reported and stored in liquid nitrogen at −80°C [14,15]. All patients were followed up for 5 years to obtain their survival information.

**Table 1.** Association between patients’ clinical features and RMRP expression

	Total (n = 118)	RMRP expression		P value
		Low(n = 56)	High (n = 62)	
Age				0.473
≤ 60	44	19	25	
>60	74	37	37	
Sex				0.504
Male	67	30	37	
female	51	26	25	
Differentiation				0.259
Well-moderate	76	39	37	
Poor	42	17	25	
Lymph node metastasis				0.027
Negative	59	34	25	
Positive	59	22	37	
TNM stage				0.003
I–II	59	36	23	
III–IV	59	20	39	

### Cell culture

Four ESCC cell lines (YES-2, KYSE30, EC109, and EC9706) and a normal esophageal epithelial cell line HET-1A were provided by the Cell Bank of Type Culture Collection of the Chinese Academy of Science (Shanghai, China). All cells were cultured in a DMEM medium with 10% FBS and 1% antibiotics. Cells were incubated in a humidified atmosphere with 5% CO_2_ at 37°C.

### Cell transfection

To regulate the expression of RMRP and NRP2, the cultured cells were transfected with RMRP small interfering RNA (siRNA, 5ʹ- CCUAGGCUACACACUGAGGACUTT-3ʹ) or pcDNA3.1-NRP2 by Lipofectamine 2000 (Invitrogen, USA) according to the manufacturer’s instruction, and untransfected cells set as the control. Transfected cells were available for the following experiments after 48 h of transfection.

### Real-time quantitative PCR (RT-qCR)

Total RNA was isolated from collected tissues and cultured cells with the help of TRIzol reagent (Invitrogen, USA). cDNA was generated by the PrimeScript 1st Strand cDNA synthesis kit (Takara, Japan) from extracted RNA. The expression of RMRP and NRP2 was evaluated by qPCR with GADPH as the internal standard and the 2^–ΔΔCT^ method as the calculated method [16]. The sequences of primer used were as follows: RMRP forward 5 ʹ – ACTCCAAAGTCCGCCAAGA-3ʹ, RMRP reverse ʹ-TGCGTA ACTAGAGGGAGCTGAC-3ʹ; NRP2 forward 5ʹ-CTGGAAGTCAGCACTAATGGAGAG-3ʹ, NRP2 reverse 5ʹ-GCATCGTTGTTGGCTTGAAATACC-3ʹ; GADPH forward 5ʹ-GGGAGCCAAAAGGGTCAT-3ʹ, GADPH reverse 5ʹ-GAGTCCTTCCACGATACCAA-3ʹ.

### Western blotting

The protein level of NRP2 was evaluated by the western blot analysis [17]. The total protein was isolated from cells by the BCA assay (Beyotime, China). The cell lysate was mixed with the SDS-polyacrylamide electrophoresis and transferred to the PVDF membrane (Millipore, USA). Then, the membranes were blocked with 5% nonfat milk followed by incubating with the anti-NRP2 (Abcam, UK) for 1 h. The secondary antibody (Amersham Pharmacia Biotech, UK) was applied and incubated for another 1 h. The protein bands were detected using the ECL luminescence kit (Pierce, USA) and quantitated with ImageJ 1.8.

### CCK8 assay

The CCK8 assay was used to evaluate the proliferation of ESCC cells according to previous studies [18]. A total of 5 × 10^4^ cells were added to each well of a 96-well plate with DMEM medium and incubated at 37°C with 5% CO_2_. CCK-8 kit was added after 0, 24, 48, and 72 h of incubation and continued incubating for another 60 min. The absorbance at 450 nm was detected with a microscope to evaluate the proliferation of cultured cells.

### Transwell assay

A total of 5 × 10^4^ cells were seeded into the upper chamber of a 24-well plate and supplied with DMEM medium and the bottom chamber was filled with DMEM medium with 10% FBS as a chemoattractant. The chambers used for invasion assay were pre-coated with Matrigel (Corning, USA). The plates were cultured at 37°C with 5% CO_2_ for 24 h. The migrated and invaded cells in the lower chamber were stained with crystal violet and counted by a microscope in triplicate [19].

### Dual-luciferase reporter assay

The dual-luciferase reporter assay was performed based on previous studies [20]. The RMRP wild type and the NRP2 wild type luciferase reporter plasmid were established by inserting the fragment containing the binding sites between miR-613 and RMRP or NRP2. The mutant plasmid was established by fragment mutation. The established plasmids were co-transfected with miR-613 mimic, miR-613 inhibitor, or negative controls. The luciferase activity was measured by the Synergy 2 Multidetection Microplate Reader (Bio Tek Instruments) normalizing to Renilla.

### Statistical analysis

Data were presented with mean value ± SD and analyzed by SPSS 23.0 and Graphpad Prism 7.0. The difference between two groups was estimated by paired Student’s t-test and the difference among multiple groups was evaluated by one-way ANOVA. The survival of patients was assessed by Kaplan-Meier analysis and the Cox regression analysis was employed to evaluate the prognostic value of RMRP. The correlation analysis was performed with the help of Pearson’s correlation analysis. *P* < 0.05 was considered to be statistically significant. All experiments were repeated at least three times independently.

## Results

RMRP was speculated to participate in the tumor progression of ESCC and be associated with the clinical outcome of patients. The specific role of RMRP and the potential mechanism were investigated with clinical tissues and *in vitro* cells.

### RMRP is upregulated in ESCC

**Figure 1.**
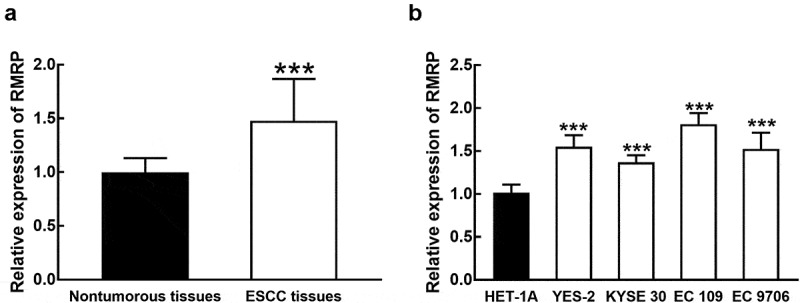
The expression of RMRP in ESCC. **A**. RMRP was significantly upregulated in ESCC tissues compared with that in adjacent nontumorous tissues. **B**. The expression of RMRP was dramatically higher in ESCC cell lines (YES-2, KYSE30, EC 109, and EC9706) than that in the normal esophageal epithelial cell line (HET-1A). ***P < 0.001

Compared with collected normal tissues, the expression of RMRP was significantly upregulated in ESCC tissues (*P* < 0.001, Figure 1a). The average expression of RMRP in ESCC tissues was used as the cutoff to divide patients into a high RMRP group (n = 62) and a low RMRP group (n = 56). Consistently in ESCC cell lines, the expression of RMRP was notably higher than that in normal esophageal epithelial cell line HET-1A (*P* < 0.001, Figure 1b).

### RMRP was associated with the clinical features and prognosis of ESCC patients

The clinical features of recruited patients were summarized in Table 1. The patients with high RMRP expression showed positive lymph node metastasis status and advanced TNM stage. With the help of the χ^2^ test, a significant association of RMRP expression with lymph node metastasis (*P* = 0.027) and TNM stage (*P* = 0.003) of patients was found.

**Figure 2.**
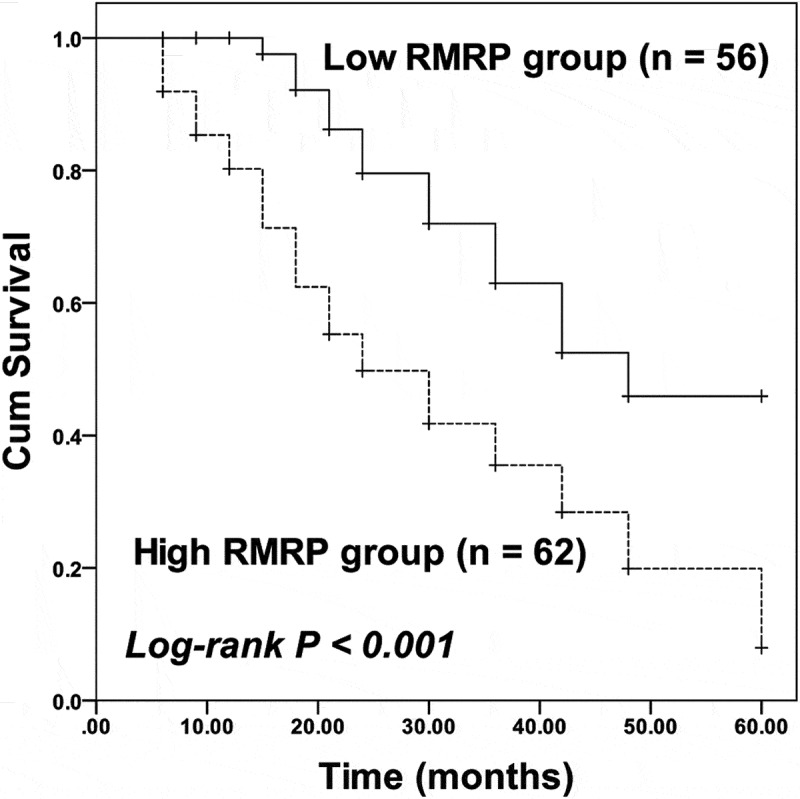
Kaplan-Meier curve of ESCC patients with different RMRP expressions. Patients with high RMRP expression possessed poorer survival than patients with low RMRP expression. Log-rank *P* < 0.001

For the survival of patients, the high RMRP was related with the poor prognosis of patients, while the low RMRP expression was associated with a better outcome of patients (log-rank *P* < 0.001, Figure 2). Moreover, RMRP (HR = 2.880, 95% CI = 1.511–5.490, *P* = 0.001) and TNM stage (HR = 1.793, 95% CI = 1.034–3.109, *P* = 0.038) were identified as independent prognostic indicators of ESCC with the employment of multivariate Cox regression analysis (Table 2).

**Table 2.** Multivariate Cox regression analysis for ESCC patients

	HR	95% CI	P value
RMRP	2.880	1.511–5.490	0.001
Age	1.268	0.721–2.231	0.409
Sex	1.128	0.648–1.962	0.670
Differentiation	1.442	0.852–2.440	0.173
Lymph node metastasis	1.549	0.903–2.659	0.112
TNM stage	1.793	1.034–3.109	0.038

### RMRP was involved in the progression of ESCC cells

**Figure 3.**
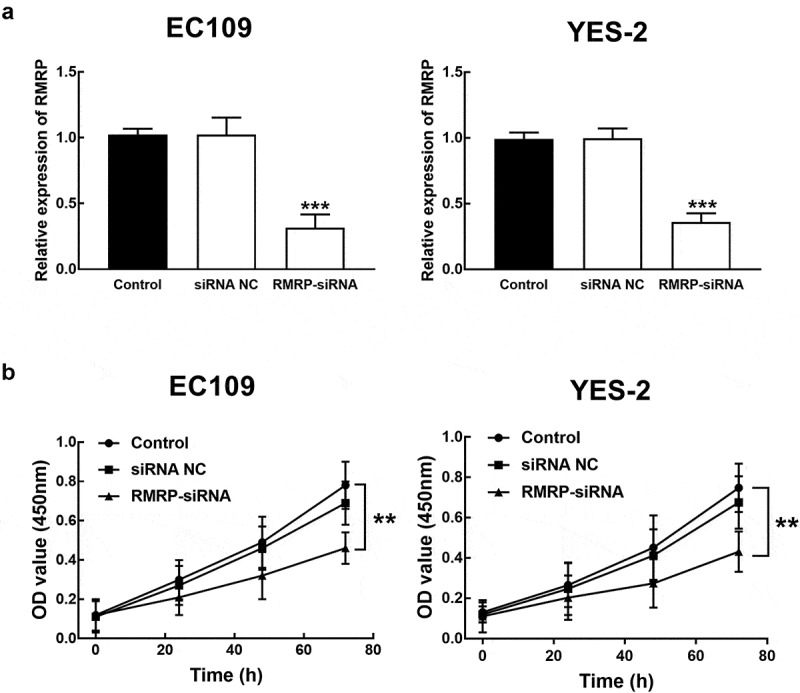
Effect of RMRP expression on cell proliferation of ESCC. **A**. The expression of RMRP in YES-2 and EC109 cells was significantly inhibited by the transfection of RMRP-siRNA. **B**. The knockdown of RMRP dramatically suppressed the proliferation of YES-2 and EC109 cells. **P < 0.01, ***P < 0.001

**Figure 4.**
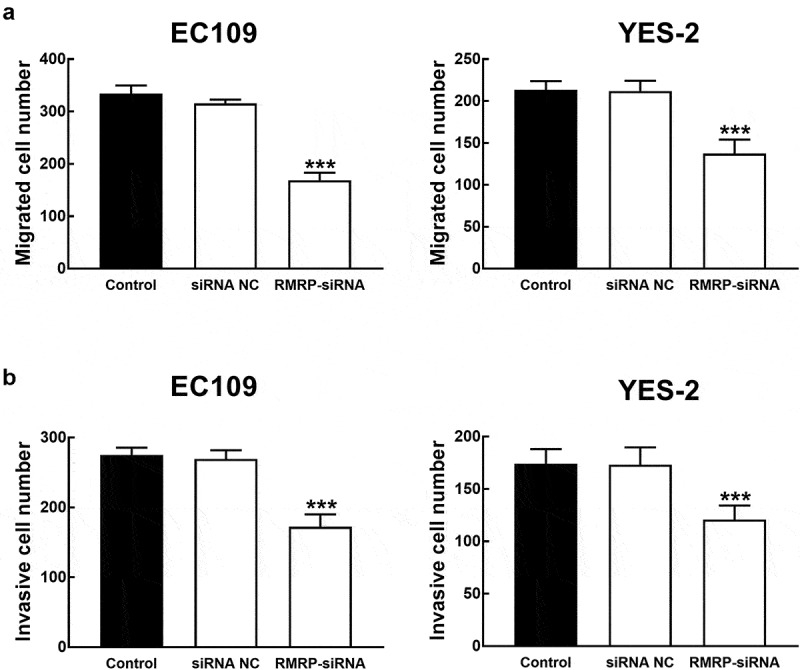
Effect of RMRP expression on cell migration and invasion of ESCC. **A**. The migration of YES-2 and EC109 cells was significantly inhibited by the knockdown of RMRP. **B**. The invasion of YES-2 and EC109 cells was significantly inhibited by the silencing of RMRP. ***P < 0.001

ESCC cells were transfected with RMRP-siRNA to silence RMRP. The expression of RMRP was significantly inhibited by the transfection of RMRP-siRNA in YES-2 and EC109 cells (*P* < 0.001, Figure 3a). The effect of RMRP expression on the proliferation of YES-1 and EC109 cells was further evaluated by the CKK8 assay, and it was found that the proliferation of YES-2 and EC109 cells was significantly inhibited by the silencing of RMRP (*P* < 0.01, Figure 3b). Similarly, in the migration and invasion of ESCC cells, the knockdown of RMRP showed a notably inhibitory effect, indicating the involvement of RMRP in the biological function of ESCC cells (*P* < 0.001, Figure 4a and b).

### RMRP regulated the miR-613/NRP2 axis

**Figure 5.**
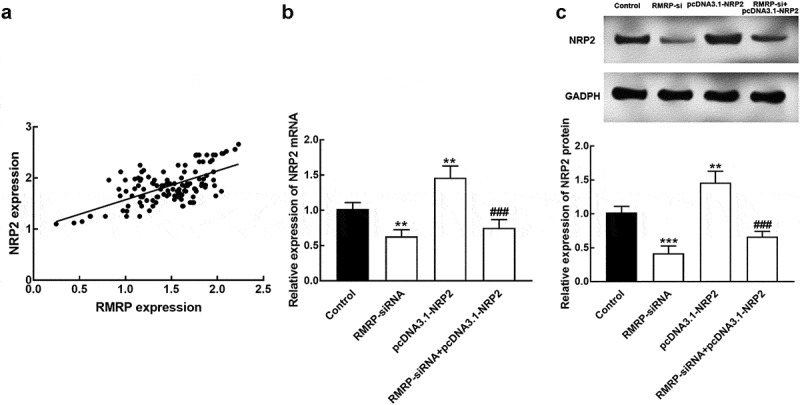
The interaction between RMRP and NRP2. **A**. The expression of RMRP was positively correlated with NRP2 expression. *r* = 0.637, *P* < 0.001. **B**. The expression level of NRP2 mRNA was significantly suppressed by the knockdown of RMRP and enhanced by the transfection of pcDNA3.1-NRP. The silencing of RMRP could reverse the overexpression of NRP2. **C**. The expression level of NRP2 protein was dramatically inhibited by the silencing of RMRP and promoted by pcDNA3.1-NRP2. The knockdown could attenuate the upregulation of NRP2. **P < 0.01, ***P < 0.001 relative to the control group. ^###^*P* < 0.001 relative to pcDNA3.1-NRP2 group

The expression of RMRP was positively correlated with the NRP2 expression level with the correlation coefficient value of 0.637 (*P* < 0.001, Figure 5a). The expression of NRP 2 mRNA decreased in the presence of RMRP downregulation and increased in the presence of pcDNA 3.1-NRP2 compared with controls (*P* < 0.01, Figure 5b). Consistently, the expression level of NRP2 protein was also inhibited by the knockdown of RMRP and enhanced by pcDNA-NRP2 (*P* < 0.01, *P* < 0.001, Figure 5c). While the co-regulation of RMRP and NRP2 could reverse the overexpression of NRP2 (*P* < 0.001, Figure 5b and c).

**Figure 6.**
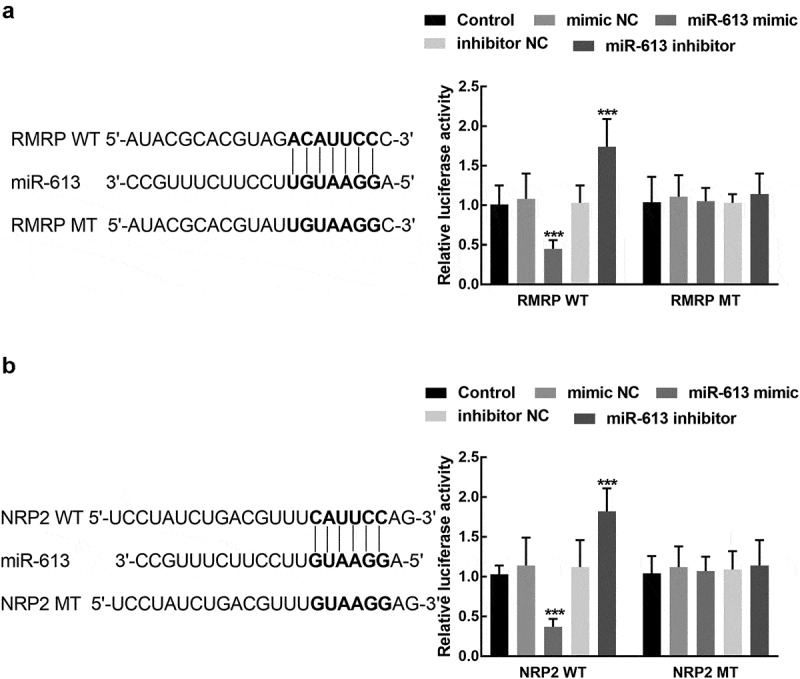
The association of miR-613 with RMRP and NRP2. **A**. miR-613 overexpression significantly suppressed the luciferase activity of RMR WT, which was enhanced by the knockdown of miR-613. **B**. miR-613 dramatically regulated the luciferase activity of NRP2 WT, while the NRP2 MT was not affected by miR-613. ***P < 0.001

The luciferase activity of RMRP was significantly suppressed by the overexpression of miR-613 and enhanced by the silence of miR-613 (*P* < 0.001, Figure 6a). For the association between miR-613 and NRP2, the results of the luciferase reporter showed that the upregulation of miR-613 dramatically reduced the luciferase activity of NRP2, which was elevated by the knockdown of miR-613 (*P* < 0.001, Figure 6b). Both the luciferase activity of RMRP MT and NRP2 MT was not affected by the regulation of miR-613 (*P* > 0.05, Figure 6a and Figure 6b).

**Figure 7.**
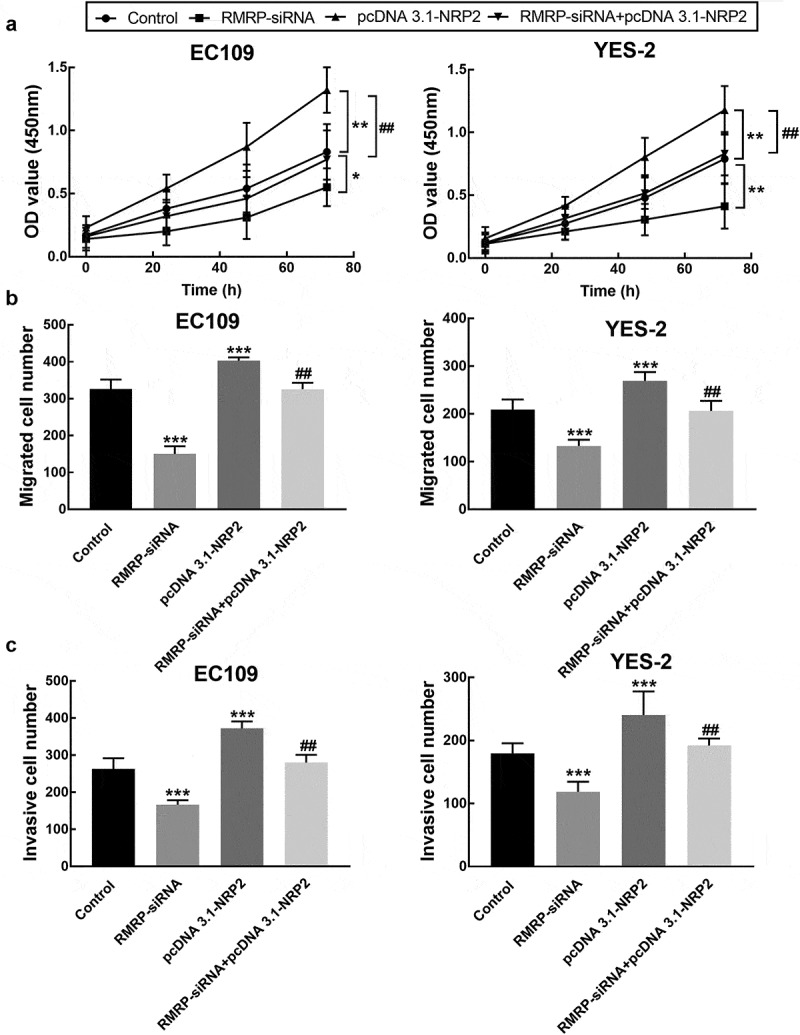
Effect of NRP2 on cell processes of ESCC. **A**. The overexpression of NRP2 significantly promoted the proliferation of EC109 and YES-2 cells, which was reversed by the knockdown of RMRP. **B**. The migration of EC109 and YES-2 cells was dramatically improved by NRP2 overexpression, which was reversed by the silencing of RMRP. **C**. The invasion of EC109 and YES-2 cells was dramatically improved by NRP2 overexpression, which was reversed by the silencing of RMRP. **P < 0.01, ***P < 0.001 relative to the control; ^##^ *P* < 0.01 relative to pcDNA3.1-NRP2

The proliferation of EC109 and YES2 cells was promoted by the overexpression of NRP2, which was reversed by the downregulation of RMRP (*P* < 0.05, *P* < 0.01, Figure 7a). While the enhancement by NRP2 upregulation was also observed in the migration and invasion of EC109 and YES2 cells, and the knockdown of RMRP dramatically alleviated the promoted effect of NRP2 (*P* < 0.01, *P* < 0.001, Figure 7b and c).

## Discussion

ESCC is one of the most common deadliest tumors worldwide with increasing new cases and mortality [21]. Novel prognostic markers and therapeutic targets are lacked, which is crucial for the prevention of oncogenesis and successful therapy. LncRNAs are important members of the noncoding RNA family, which have been discovered to affect epigenetic characteristics and provide cellular information for tumor progression [22]. There are a number of molecules, including lncRNAs, have been appraised as effective biomarkers to predict clinical prognosis and disease development [23,24]. For example, lncRNA LOXL1-AS1 facilitated the progression of pancreatic cancer through regulating miR-28-5p and SEMA7A [25]. LncRNA-D16366 was identified as a diagnostic and prognostic indicator of hepatocellular carcinoma due to its close relationship with the early detection and risk prediction of hepatocellular carcinoma patients [26]. RMRP is a lncRNA located on 9p13.3 which has attracted special attention in recent studies focused on tumor development.

In this study, the expression of RMRP was examined in ESCC patients and found that RMRP exhibited significantly higher expression in ESCC tissues and cell lines than normal tissues and esophageal epithelial cells. The upregulation of RMRP was closely associated with the poor outcome of patients. The dysregulation of RMRP implies that it may participate in the development of ESCC. The high expression of RMRP also showed a close relationship with the lymph node metastasis and TNM stage of patients, which are critical indicators to reflect the deterioration of patients, suggesting the potential role of RMRP in the disease progression of ESCC. RMRP was reported to be upregulated in a wide range of human diseases, such as cardiac fibrosis, myocardial ischemia-reperfusion injury, and lung cancer [27–29]. RMRP has been reported to be involved in the development of various cancers. Cao et al. revealed the enhancement of RMRP on the proliferation, migration, and invasion in bladder cancer [6]. In glioma, RMRP was associated with the tumor grade and Karnofsky Performance Scores of patients and therefore contributed to the progression of glioma [30]. Here, RMRP was also found to regulate the biological function of ESCC cells. The knockdown of RMRP significantly inhibited cell proliferation, migration, and invasion of ESCC cells, which is consistent with previous studies [30–32].

NRP2 is one of the major isoforms of NRPs, which are non-tyrosine kinase receptors [33]. In previous studies, the cellular functions of NRP2 have been disclosed in various cancers. For example, in papillary thyroid cancer, NRP2 could promote cell growth and is closely associated with extrathyroid extension and lymph node metastasis [34]. It was also reported that NRP2 could act as an independent prognostic factor for prostate adenocarcinoma patients, as its upregulation was correlated with the worse outcome of patients [35]. The tumor promoter role of NRP2 in ESCC has been demonstrated in our previous study, and a positive correlation between RMRP and NRP2 was found in the present study [13]. Furthermore, the interaction between RMRP and NRP2 was validated in the present study. RMRP was found to positively regulate the expression of NRP2 and its knockdown could reverse the overexpression of NRP2 by the transfection of pcDNA3.1-NRP2. Additionally, RMRP was revealed to sponge miR-613, which regulated the expression of NRP2. Meanwhile, the knockdown of RMRP could reverse the promoted effect of NRP2 in cell proliferation, migration, and invasion of ESCC. Therefore, regulating the miR-613/NRP2 axis was speculated to be the mechanism underlying the functional role of RMRP in ESCC.

However, the specific mechanism underlying the interaction between RMRP and miR-613/NRP2 axis has not been validated *in vivo* in this study. While due to the relatively small sample size, the clinical significance of RMRP may not be excavated entirely. Therefore, further investigations focused on the underlying mechanism with a larger sample size are needed.

## Conclusion

Taken together, the upregulation of RMRP and its close association with the poor outcome of ESCC was revealed in this study. RMRP might serve as a tumor promoter to accelerate cell proliferation, migration, and invasion of ESCC through regulating the miR-613/NRP2 axis.
